# Beneficial Phytochemicals with Anti-Tumor Potential Revealed through Metabolic Profiling of New Red Pigmented Lettuces (*Lactuca sativa* L.)

**DOI:** 10.3390/ijms19041165

**Published:** 2018-04-11

**Authors:** Xiao-Xiao Qin, Ming-Yue Zhang, Ying-Yan Han, Jing-Hong Hao, Chao-Jie Liu, Shuang-Xi Fan

**Affiliations:** 1 Beijing Key Laboratory of New Technology in Agricultural Application, National Demonstration Center for Experimental Plant Production Education, Beijing Collaborative Innovation Center for Eco-Environmental Improvement with Forestry Fruit Trees, Plant Science and Technology College, Beijing University of Agriculture, Beijing 102202, China; vipqindada@163.com (X.-X.Q.); m18813014019@163.com (M.-Y.Z.); hyybac@126.com (Y.-Y.H.); haojinghong@126.com (J.-H.H.); cliu@bua.edu.cn (C.-L.L.); 2 Beijing Bei Nong Enterprise Management Co., Ltd., Beijing 102206, China

**Keywords:** polyphenols, red pigmented lettuce, antioxidant activity, anti-tumor effects

## Abstract

The present study aimed to compare polyphenols among red lettuce cultivars and identify suitable cultivars for the development and utilization of healthy vegetables. Polyphenols, mineral elements, and antioxidant activity were analyzed in the leaves of six red pigmented lettuce (*Lactuca sativa* L.) cultivars; thereafter, we assessed the anti-tumor effects of cultivar B-2, which displayed the highest antioxidant activity. Quadrupole–Orbitrap mass spectrometry analysis revealed four classes of polyphenols in these cultivars. The composition and contents of these metabolites varied significantly among cultivars and primarily depended on leaf color. The B-2 cultivar had the highest antioxidant potential than others because it contained the highest levels of polyphenols, especially anthocyanin, flavone, and phenolic acid; furthermore, this cultivar displayed anti-tumor effects against the human lung adenocarcinoma cell line A549, human hepatoma cell line Bel7402, human cancer colorectal adenoma cell line HCT-8, and HT-29 human colon cancer cell line. Hence, the new red-leaf lettuce cultivar B-2 has a distinct metabolite profile, with high potential for development and utilization of natural phytochemical and mineral resources in lettuces and can be used as a nutrient-dense food product.

## 1. Introduction

Lettuces, family Asteraceae (Composite), are the most popular vegetables either used as ingredients for salads or consumed fresh owing to beneficial health effects [1,2]. Iceberg and butterhead lettuce are the most popular cultivars predominantly used for preparing salads, and the demand for green and red oak-leaf lettuces has considerably increased in recent years [3]. Lettuce of three different colors can be obtained: dark-red, red, and green; in recent years, red lettuce is commonly used in salads or regularly consumed raw because of its attractive red color and appealing taste [4]. Red lettuce has strong antioxidant activity owing to its higher anthocyanin content than that of green lettuce; moreover, red vegetables and fruits usually have greater health benefits in humans [5,6,7,8]. The increased demand of “health food” associated with high antioxidant potential and reduced risk of diseases has resulted in increased consumerism worldwide; moreover, the quality, quantity, and wide variety of healthy food products are essential for the market. Various approaches involving cultivars, including cultural and management related practices are important to enhance the quality of lettuce; in particular, a wide variety would broaden the phytochemical spectrum and other health-promoting attributes [9,10,11].

Anthocyanins are water-soluble phenolic glycosides derived from anthocyanidins; many fruits, vegetables, and pigments have color owing to different types of anthocyanidins such as cyanidin, delphinidin, pelargonidin, and malvidin [12,13]. The free radical-scavenging potential of anthocyanidins and its anti-inflammatory effects have been reported. Cyanidin-3-*O*-glucoside has been reported to display anti-tumor activity in breast cancer [14]. In addition to being rich in anthocyanidins, red lettuce is also a good source of different classes of phenolic compounds, primarily hydroxycinnamic acid, usually from caffeic acid derivatives and flavonols [10,15]. Dark vegetables have higher nutrient composition, anthocyanin content, vitamin C, and mineral content than green vegetables, especially purple cabbage, red pepper, and red lettuce [16,17,18]. The nutrient composition of vegetables is an important index for studying their health benefits, and studies on red lettuce as a new cultivar are increasing [19]. Biological functions of nutrients have increased the current interest in developing healthy food products, and consumers have rendered vegetables as an important source of antioxidants in their daily diet. Simultaneously, studies have emphasized that the choice of cultivar determines the nutritive index and health benefits; hence, strategizing the selection of the appropriate cultivar is critical to maximally increase levels of bioactive nutrients [20,21].

In this study, based on previous findings obtained from breeding red lettuces in our laboratory and a comprehensive evaluation of vegetable shape, taste, and yield, we investigated the nutrient profiles of six red lettuce cultivars and investigated their nutritional quality and biological function. We attempted to identify the important metabolites in red lettuce and mineral elements in different cultivars, and compared the antioxidant capacity of different cultivars based on the 2,2′-azino-bis(3-ethylbenzothiazoline-6-sulfonic acid) (ABTS), 2,2-diphenyl-1-picrylhydrazyl (DPPH), and total antioxidant capacity (T-AOC-FRAP) antioxidant indices. Furthermore, we used a 3-(4,5-dimethylthiazol-2-Yl)-2,5-diphenyltetrazolium bromide (MTT) assay to determine the effect of red lettuce on cancer cell growth. This study aimed to compare polyphenol levels in lettuce cultivars and identify suitable cultivars for the development and utilization of healthy vegetables. Our results may help understand the nutrient profile of red lettuce and provide a technical theoretical support for cultivators and consumers to choose lettuce varieties with higher nutritional value.

## 2. Results and Discussion

### 2.1. Phytochemical Composition in Different Red Lettuce Cultivars

**Figure 1:**
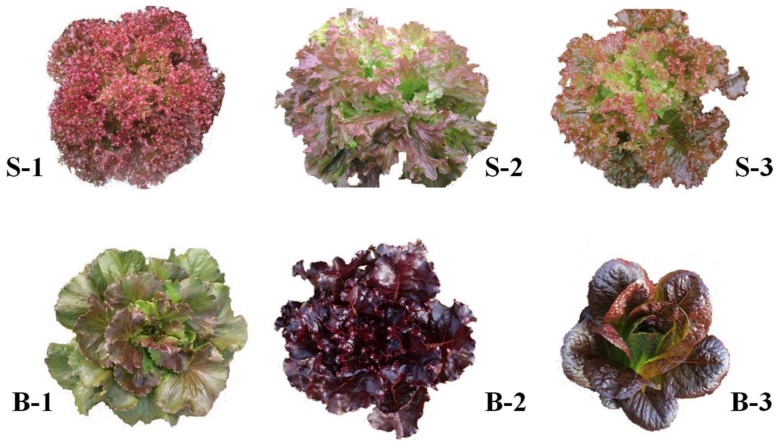
Morphological characterization of the six red lettuce cultivars.

We harvested and extracted whole edible leaves from six red-lettuce cultivars (Figure 1), including two dark-red lettuce cultivars (B-2 and B-3) and four red lettuce cultivars (S1, S2, B-1, and S-3). The phytochemical composition was determined through ultra-high performance liquid chromatography with a diode-array detector with electrospray ionization and quadrupole time-of-flight mass spectrometry in tandem mass spectrometry MS^E^ mode (UHPLC-DAD-ESI-QTOF/MS) analysis and in accordance with previously reported data on fragmentation patterns [15]. Phytochemical data are presented in Table 1, and the tentatively identified compounds included phenolic acids, anthocyanin, flavonol, sesquiterpenoids, and other phenylpropanoids. These compounds were considered the primary healthy components [22]. 

To obtain an overview of the distribution of the six samples and highlight discriminant analytes, the data indicating significant findings on analysis of variance (ANOVA) were further explored using orthogonal partial least squares–discriminate analysis (OPLS-DA). Variables with high relevance for explaining the differences among cultivars were identified. A total of 71 main phytochemicals were identified (Table 1), and the variable importance for the projection (VIP) score indicated the preliminary screening of the metabolites in different cultivars; furthermore, we could combine the p-value or fold change of the VIP to represent the great differences in metabolites. The retention time, accurate mass, fragment ions, and VIP are listed in Table 1, 30 significant differences among six cultivars (VIP > 1) are displayed. The tentatively identified compounds included organic acids, vitamins, phenolic acids, and flavonoids. The assigned compounds with high VIP values (VIP > 2) were anthocyanin, flavone, and phenolic acid. These compounds were considered to be the most contributed features for health benefits in red-lettuce cultivar, and the content (semi-quantitative information) of these phenolic compounds are listed in Table S1.

**Figure 2:**
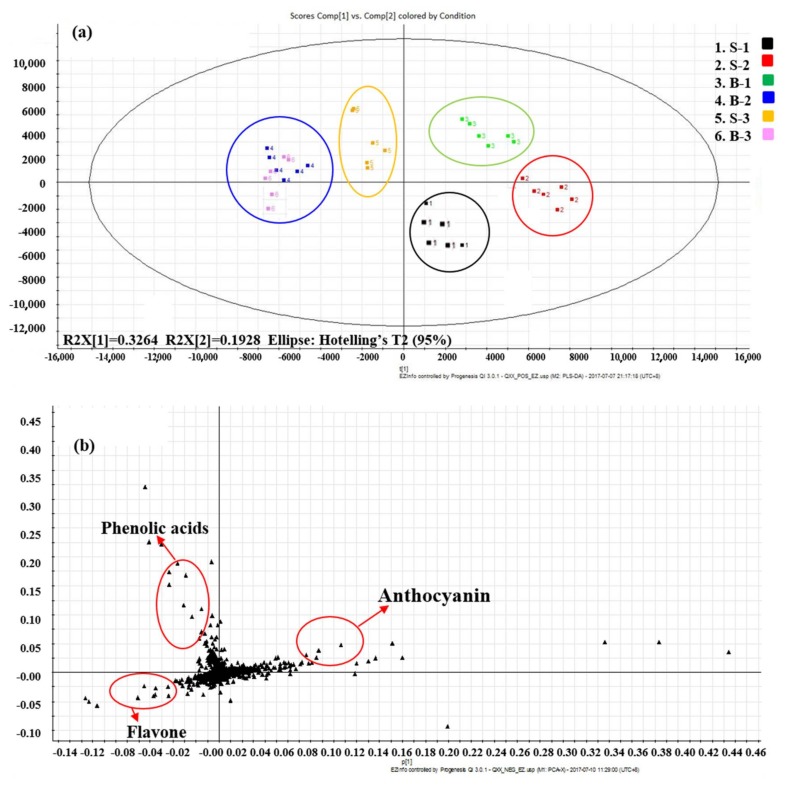
Orthogonal partial least squares–discriminate analysis (OPLS-DA) of six red-lettuce cultivars analyzed via UHPLC–Quadrupole–Orbitrap MS/MS (every cultivar included six independent biological replicates). (**a**) Score plot. (**b**) Loading plot.

The scope plot of the OPLS-DA (Figure 2a) revealed a clear separation among cultivars. Essentially, the six cultivars were separated in different quadrants. B-2 and B-3 were located close to the second quadrant, and they had similar metabolite profiles; the other cultivars displayed different metabolite compositions. When comparing these metabolites among the aforementioned cultivars, anthocyanin, phenolic acid, and flavone levels served as prominent distinguishing factors among different red-lettuce cultivars (Figure 2b, Table S1). Lettuce contains several types of phenolic compounds, hydroxycinnamic acids being the primary phenolic compounds; furthermore, other caffeic acid derivatives and glycosylated flavonoids have been reported, such as coumaroylquinic acid, chlorogenic acid, caffeoylmalic acid, cyanidin, quercetin, kaempferol, and luteolin derivatives, and some of them are related to specific cultivars [1,23,24,25,26,27]. Furthermore, the polyphenol content in lettuce tissues are various among cultivars and growing conditions, and anthocyanins have only been detected in red cultivars [3,28,29,30].

### 2.2. Mineral Content in Different Red Lettuce Cultivars

Ca, Cu, Fe, K, Mg, Mn, Na, P, and Zn were detected and their contents differed among cultivars (Table 2). Vegetables normally contribute nutrients to humans through their daily dietary intake; hence, diets deficient in vitamin and mineral absorption induce nutritional disorders. In the present study, differences in leaf mineral concentrations were primarily dictated by the species during harvesting and different types of mineral components showed significant differences in the same cultivar. Across species, K was the predominant macronutrient (mean content of 42.7083–55.8750 mg/g DW; Table 2), and Cu was of the least predominant (mean content of 0.0148–0.0157 mg/g DW; Table 2). All leafy vegetables contained high levels of minerals, particularly B-1, B-2, and S-1 cultivars. Specifically, the highest P and K contents were observed in B-1; the highest Na content, B-1 and B-2; highest Ca and Mg content, S-3 and B-4. Other minerals including Cu, Fe, Mn, and Zn showed no significant difference. Our results indicate the composition of various elements and their content in six red lettuce leaves; compared to those of lettuces reported previously, the six cultivars had a higher mineral composition and hence a greater health benefit [31]. Differences in mineral composition reported previously could be attributed to cultivars, different farming practices, and environmental conditions [11]. In the present study, disparities in mineral content were primarily influenced by the cultivars.

### 2.3. Comparison of Antioxidant Activity in Different Red-Lettuce Cultivars

Owing to the complex reactivity of phytochemicals, the antioxidant activities of natural matrices and food extracts cannot be evaluated by a single method; however, at least two test systems have been recommended for the determination of antioxidant activity. Hence, the antioxidant activity of samples was determined using three spectrophotometric methods: DPPH, ABTS, and T-AOC-FRAP analyses. B-2 and B-3 lettuce extracts possessed the strongest antioxidant activity when compared with the others cultivars and S-2 antioxidant capacity was the lowest, while that of S-1 was similar to that of B-2 and B-3, and that of S-3 is higher than that of B-1 (Table 3). The reduction of DPPH absorption indicates the capacity of the samples to scavenge free radicals, whereas the ABTS and T-AOC-FRAP method is used to determine the capacity of reductants in a sample. DPPH analysis revealed higher free radical scavenging activity of lettuce extracts than that of ABTS and T-AOC-FRAP analyses. Our preliminary studies revealed that anthocyanin and flavonoid contents followed the following pattern in the leaves: B-2 > B-3 > S-1 > S-3 > S-2 > B-1 [32]. 

These results may be attributed to the fact that the B-2 and B-3 red-lettuce cultivars are rich in anthocyanin, especially in cyanidin derivatives, and to the presence significance differences in phenolic acid and flavonol levels, which contribute to the antioxidant activity, since anthocyanin, phenolic acid, and flavonols have free radical scavenging activity [1,29,33,34].

### 2.4. Analysis of In Vitro Anticancer Effects Activity in the New Dark-Red Lettuce Cultivar (B-2)

**Figure 3:**
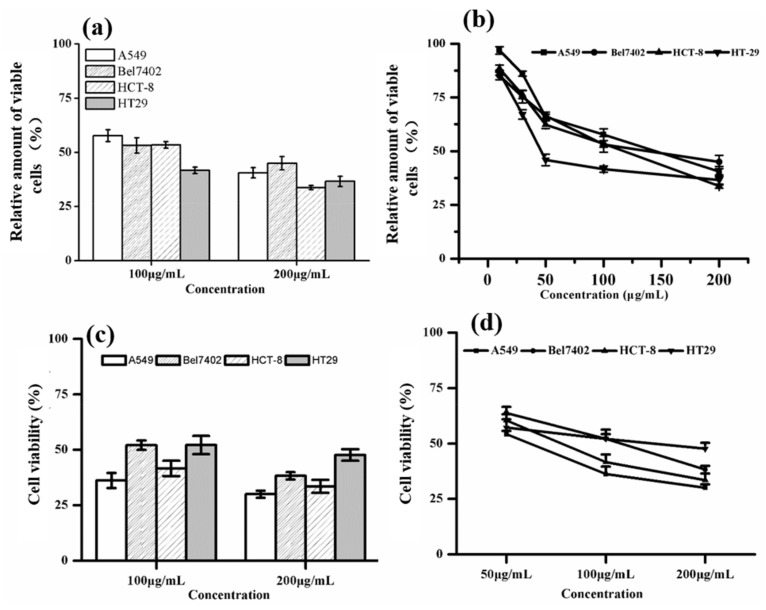
Anti-tumor therapeutic effects of the B-2 lettuce. (**a**,**b**) Relative amount of viable cells assayed by Thiazolyl Blue Tetrazolium Bromide (MTT); (**c**,**d**) cell viability assayed by trypan blue staining. Human lung adenocarcinoma cell line A549, human hepatoma cell line Bel7402, human cancer colorectal adenoma cell line HepG2, and colon cancer cell line HT-29 were treated with the indicated concentrations of extracted fractions of B-2. The mean ± SD values of six separate experiments are shown.

B-2 had the rich phytochemical profile and higher mineral element content, and the highest antioxidant activity among the six cultivars. To identify nutrient-rich cultivars with potential application as nutrient-dense food products, we selected the B-2 to investigate its in vitro anticancer effects. We chose ethanol as the extracting solution due to the fact that it is cheap, non-toxic, and suitable for large-scale production extraction. The relative amount of viable cells are shown in Figure 3a,b, when the B-2 extract was administered at 100 μg/mL, it decreased the relative amount of four viable cancer cell lines, when the extract up to the concentration of 200 μg/mL, it inhibited the relative amount of viable cells in a more robust manner (Figure 3a). Moreover, different tumor cells displayed different sensitivities to the extract, and in our study, when the concentrations of B-2 extracts increased, the relative amount of viable of the four tumor cell lines were decreased (Figure 3b). The MTT assay data indicated that the B-2 extract could decrease the viable amount of the four tumor cell lines.

The trypan blue (TB) assay showed tumor cells viability (Figure 3c,d), when the B-2 extract was administered at 100 μg/mL, it decreased tumor cell viability; at 200 μg/mL, tumor cell viability decreased further (Figure 3c). Furthermore, different tumor cells displayed different sensitivity to the extract, and in our study, when the concentrations of B-2 extracts increased, the viability of the four tumor cell lines decreased (Figure 3d). TB assay data indicate that the B-2 extract could decrease the tumor cell viability.

Reported concentrations of total anthocyanidin metabolites in human plasma after consumption of red wine (20.0–35.0 mg/100 mL) range from 0.01 to 0.11 μM, flavanones metabolites in human plasma after consumption of orange juice (15.0 mg/100 mL) range from 0.06 to 0.64 μM, and grapefruit metabolites (54.5 mg/100 g) range from 5.99 μM [35,36,37]. Plasma levels of flavonoids in humans generally peak between 1 and 3 h after consumption of flavonoid-rich foods, are between 0.06 and 7.6 μM for flavonols, flavanols, and flavanones, and less than 0.15 μM for anthocyanidins. Six men and five women consumed 250 g of fresh lettuce, and blood was sampled before and 2, 3, and 6 h after consumption. Plasma antioxidant capacity was measured and total radical-trapping antioxidant parameter (TRAP) increased by 40–50%, which was associated with significantly increased plasma levels of quercetin, *p*-Coumaric acid, and vitamin C [38]. Low levels of flavonoids and their metabolites probably exert other biological effects, e.g., altered cell signaling and gene expression, which contribute to their purported health benefits. Considering the anticancer activity of cultivar B-2, it should be noted that, per the NCI (National Cancer Institute, Bethesda, MD, USA), a crude plant extract is promising for further purification when its IC_50_ value is lower than 30 μg/mL, when attempting to identify potential natural anticancer compounds [39]. Although the in vitro anticancer effects of B-2 crude extract were observed at a concentration greater than 30 μg/mL, since red-lettuce is directly edible, it can be an important functional food for tumor prevention. Our results showed the dietary significance of this inexpensive and popular lettuce for human body health; furthermore, our study can help identify nutrient-rich cultivars with potential applications.

## 3. Materials and Methods 

### 3.1. Plant Materials

The leaves of leaf red-lettuce (*Lactuca sativa* L.) cultivars marked S-1, S-2, B-1, B-2, S-3, and B-3 were grown in the Changping District of Beijing Seed Management Experimental Station under standard greenhouse conditions. S-3 and B-3 are the control varieties, as they are widely available in the market and other varieties were bred in our laboratory. Peat control and water management were performed in accordance with standard practice, with temperature control at 20–25 °C in the daytime and 10–15 °C at night. The six cultivars were planted on August 2017 and harvested on November 2017. The experiment was performed using a randomized block design of six cultivars with three biological replicates, and 5–10 were used in each plot. After collection of their leaves per plot, freezing them in liquid nitrogen, and storing them at −80 °C, mass spectrometry (MS) analysis and analysis of in vitro anticancer effects activity were performed. The remaining leaves were stored at −20 °C for enzyme analysis.

### 3.2. UPLC–QTOF-MS Analysis Phytochemical Composition

Some fresh lettuces were placed in a ModulyoD-230 freeze dryer (Thermo Fisher, New York, NY, USA) to obtain a powder at −80 °C, 1.0 g of dry powder was collected, and 10 mL of methanol/water/formic acid (80:19:1, *v*/*v*/*v*) was added to the sample, followed by 70 min of bath sonication at 12,000 Hz at 45 °C to extract phytochemicals. The compounds were filtered through a 0.22 μm membrane (Shanghai ANPEL, Shanghai, China) before ultra-performance liquid chromatography (UPLC)-MS analyses.

An ACQUITY UPLC I-Class with FTN Sample Manger instrument (Waters, Milford, MA, USA) was used in our experiments. For chromatographic separation, Eluent A was 0.01% aqueous formic acid, and Eluent B was 100% acetonitrile. Separation was performed using the following elution gradient: 90% A at 0 min, 90% A to 81% A from 0 to 3 min, 81% A to 70% A from 3 to 4 min, 70% A to 60% A from 4 to 5 min, 60% A to 5% A from 5 to 7 min, and 5% A to 90% A from 7 to 9 min. The flow rate was 0.4 mL/min, and 2 μL aliquots of the analytes were injected. The column temperature was maintained at 30 °C for all analyses. The photodiode array detector functioned between 200 and 600 nm.

Xevo G2-S QTOF (Waters MS Technologies, Milford, MA, USA), a quadrupole and orthogonal acceleration time-of-flight tandem mass spectrometer, was used with an electrospray ionization source. Both positive and negative ion modes were used for compound ionization. Mean square error data were collected. At one sample injection, the mode could collect precise mass data of quasi-molecular ions and fragment ions by alternating the low and high collision energy rapidly. The detection conditions were as follows: capillary voltage, 0.45 kV; cone voltage, 40 V; source temperature, 120 °C; desolvation temperature, 500 °C; cone gas flow, 50 L/h; desolvation gas flow, 700 L/h; low energy, 6 V; high energy ramp, 20–40 V. Time-of-flight (TOF)-MS ranged from 100 to 1200 *m*/*z*. The scan time was 0.2 s. All analyses were obtained using the Lockspray to ensure accuracy and reproducibility. Leucine-enkephalin was used as the lockmass at a concentration of 200 ng/mL and a flow rate of 10 μL/min. Data were acquired in real time (scan time, 0.5 s, interval, 15 s). The UPLC-QTOF-MS data of samples were acquired and analyzed using Waters UNIFI 1.7 software (V1.7, Waters Corporation, Milford, CT, USA).

Mass spectrometry can be carried out using mass analyzers with a range of mass resolution, triple quadrupoles was capable of measuring metabolite masses with unit resolution. Triple quadrupole instruments can be used to carry out tandem mass analysis (MS/MS). Here, each quadrupole has a separate function; the first quadrupole (Q1) scans across a preset *m*/*z* range for selection of one or more ions of interest, with fragmentation in the second quadrupole (Q2) using a collision gas (argon). Q2 is typically an octapole in modern triple quadrupole instruments. According to the subsequent selected reaction monitoring (SRM) experiment, fragment ions generated in Q2 can be subjected to further selection, and this SRM capability of triple quadrupole instruments constitutes a highly sensitive approach for quantifying known metabolites.

### 3.3. Analysis of Mineral Content

Of the total freeze-dried samples, 0.2 g was used for analysis with the microwave muffle digestion system (Multiwave 3000, Anton Paar GmbH, Graz, Austria) to prepare the test solution. Thereafter, by using inductively coupled plasma atomic emission spectroscopy (ICPE-9000, Shimadzu, Kyoto, Japan), the mineral content was quantified. A total of nine elements (Ca, Cu, Fe, K, Mg, Mn, Na, P, and Zn) were analyzed. The same specimen was tested four times, with high reproducibility. For mineral quantity, the diluted standard solution was prepared by purchasing a 100 ppm standard solution (An Apex Co., Seoul, Korea), and high-purity argon gas was used. Every tool used in the experiment resulted in no contamination.

### 3.4. Analysis of Antioxidant Activity

Each sample was extracted using a methanol/water/formic acid (80:19:1, *v*/*v*/*v*) solution and a material-to-solvent ratio of 1:10 under sonication at a frequency of 12,000 Hz at 45 °C. Extraction was performed three times in total for 2.0, 1.5, and 1.0 h. Subsequently, the solutions were filtered and concentrated with a rotary evaporator at 40 °C and finally freeze-dried at −80 °C in a ModulyoD-230 freeze dryer (Thermo Fisher) to obtain sample powder. We accurately weighed 25.00 mg of the sample powder in a 25 mL brown volumetric flask separately, dissolved it in 80% methanol and adjusted the final volume to 25 mL to obtain a standard stock solution (1.00 mg/mL). A stepwise dilution method was used to configure a series of standard solutions. 

An antioxidant assay was analyzed using the method of Maria John et al. and Lee et al. [40,41] with some modifications. ABTS and DPPH powder were purchased from Sigma-Aldrich (St. Louis, MO, USA) and DPPH powder (0.0196 g) was dissolved in 500 mL of methanol and then set to 0.10 mM. Potassium peroxodisulfate solution (140 mM) was mixed with 7 mM ABTS solution (1:1, *v*/*v*) to react overnight at 25 °C in the dark to obtain ABTS radical working solution. Two concentrations of working solution were prepared, and the absorbance value was 0.70 ± 0.02 at 414 nm. The fresh ABTS radical working solutions were prepared for each assay. First, 50 μL of 0.5 mg/mL sample extracts were added to 3950 μL of radical solution (ABTS and DPPH). After 15 min, the absorbance was measured spectrophotometrically at 517 nm (DPPH)/414 nm (ABTS). DPPH/ABTS radical scavenging activity was determined using the following equation:

DPPH/ABTS scavenging effect (%) = [(A_0_ − At)/A_0_] × 100%

where At implies the absorbance in the presence of the compounds, and A_0_ is the absorbance without those compounds.

All enzymes were extracted at 4 °C. Each sample (0.1 g fresh weight) was thoroughly homogenized in 1.0 mL of 50 mM phosphate buffer (pH 7.8) containing 0.1 mM ethylene diamine tetraacetic acid (EDTA) and 0.05 g of quartz for homogenization. The homogenate for T-AOC-FRAP analysis was centrifuged at 10,000× *g* for 10 min, and T-AOC-FRAP was measured using the ferric reducing activity of plasma (FRAP) assay and commercial kits (Product Codes: FG5, Suzhou Comin Biotechnology Co., Ltd., Suzhou, China). 

### 3.5. Extraction of Phytochemical Composition in Red-Lettuce B-2 Cultivar

Ten kilograms of fresh red-lettuce leaves were ground into a slurry, using a soya-bean milk machine (Wallmate Corporation, Suzhou, China, No, TAOB-40236093317) and extracted with a hot refluxing extraction method (an 80% ethanol solution was used for extraction and extracted three times; each extraction time was 2, 1.5, and 1 h; extraction temperature was 80 °C; the solid-to-liquid ratio was 1:10, implying that 1 kg of sample were extracted with 10 L of 80% ethanol solution). Thereafter, we used cotton for vacuum filtration of the extraction solution, the flavonoids and phenolic acid were separated via AB-8 macroporous resin and, using a 95% ethanol elution, spin steaming enrichment (until the screw ethanol was evaporated out). The extracts were then dissolved with purified water, and the concentrated solutions were frozen at −80 °C with a ModulyoD-230 freeze dryer (Thermo Fisher) and reduced to a powder. 

### 3.6. Analysis of Tumor Cell Growth Inhibition In Vitro

An MTT cell viability assay was performed. Mitochondrial succinate dehydrogenase can convert the insoluble violet crystalline formazan produced from MTT and deposit it in the cells, but dead cells cannot. Dimethyl sulfoxide (DMSO) could dissolve the formazan in cells, and its light absorption value was measured by ELISA at 490 nm. The number of living cells can be determined from the measured absorbance value (OD value).

Human lung adenocarcinoma cell line A549, human hepatoma cell line Bel7402, human colorectal cancer cell line HepG2, and colon cancer cell HT29 (ATCC, Manassas, VA, USA) were used in our assay [42]. These four tumor cell lines were selected in their logarithmic growth phase with trypsin digestion and cultured in RPMI1640 Dulbecco’s modified Eagle medium (DMEM) supplemented with 10% fetal bovine serum (GIBCO BRL, Carlsbad, CA, USA) and obtained a cell suspension of 15,000 cells/mL. Thereafter, these cells were seeded in 96-well plates at 190 μL/plate at 37 °C for 24 h and 5% CO_2_. 

The samples were diluted to 10, 30, 50, 100, 200 μg/mL by normal saline in an MTT assay. A 10 μL aliquot of the test samples was added to cells and cultured at 37 °C for 3 days at 5% CO_2_. The colorimetric MTT assay was referred to as Dai [43]. The A549, Bel7402, and HepG2 cells were treated with MTT solution (a final concentration of 0.5 mg/mL in DMEM) for 4 h at 37 °C in a 96-well plate, the supernatant was carefully removed, and DMSO (150 μL) was added to each well to dissolve the precipitate. The absorbance at 570 nm was measured using a Model 680 microplate reader (BIO-RAD, Hercules, CA, USA).

The loss of membrane integrity of non-viable cells allows for the permeation of dyes such as trypan blue (TB) into the cell. Based on this principle, it is possible to determine cell viability by the capacity of a viable cell to exclude TB, and a dead cell to incorporate it [44]. Cell viability was detected with Trypan Blue staining previously reported by Sean D.A. [45].

cell viability (%) = (total number of viable cells/total number of viable cells + total number of dead cells) × 100%

### 3.7. Statistical Analysis

The MS data were pre-processed using SIEVE 2.2 software (Thermo Scientific, Waltham, MA, USA) and used for peak extraction, alignment, filtration, normalization and feature identification. OPLS-DA was performed to analyze differences in the metabolite profiles among cultivars and a VIP score greater than 1.0 was chosen to select the most discriminant features. Only features with VIP > 1 were assigned.

The data are presented as the mean ± standard deviation (SD). Analysis of variance (ANOVA, SPSS 17.0 software, SPSS Inc., Chicago, IL, USA) of all values was performed to assess differences in the means among different samples (*p* < 0.05 indicated statistical significance). Duncan’s multiple analysis and a Student’s t-test were used to identify significant differences among groups (*p* < 0.05, *p* < 0.01). Graphs were prepared in Origin Pro 8.0 SR4 (Origin Lab, Northampton, MA, USA) and Microsoft Office PowerPoint 2007.

## 4. Conclusions

This study investigated the major bioactive metabolites and antioxidant activities of six red lettuce cultivars. The qualitative and quantitative data suggest that the composition and contents of metabolites in lettuces differed significantly among cultivars and primarily depend on leaf color. The B-2 cultivar displayed potent ABTS and DPPH scavenging activity, and showed the highest total antioxidant (T-AOC-FRAP) activity, probably because of the higher of anthocyanin, flavone, and phenolic acid content. Furthermore, the significantly higher anti-tumor activity of B-2 cultivar increases its nutritional value, and its consumption can minimize the oxidative stress-related diseases. Our study thus suggests that the new red-leaf lettuce cultivar B-2 has a distinctive nutritional value and promotes various health benefits.

## Figures and Tables

**Table 1:** Main phytochemical composition identified via Quadrupole–Orbitrap MS/MS in six red-lettuce cultivars.

Compounds	Rt (min)	Molecular Weight (Da)	Ion Mode	Q1 (Da)	Q3 (Da)	Class	VIP	Semi Quantitative
Cyanidin 3-*O*-glucoside	2.58	448.3	Negative	447.3	284.1	Anthocyanin	1.07	+
Malvidin 3-*O*-glucoside (Oenin)	2.86	493.2	Positive	493.2	331.6	Anthocyanin	2.01	++
Cyanidin 3-*O*-glucosyl-malonylglucoside	3.06	697.1	Positive	697.1	686.9	Anthocyanin	2.59	++
Cyanidin-3,5-*O*-diglucoside (Cyanin)	3.17	611	Positive	611	287.7	Anthocyanin	3.47	+++
Delphinidin 3-*O*-glucoside (Mirtillin)	4.73	465.1	Positive	465.1	303.1	Anthocyanin	1.02	+
Cyanidin *O*-malonyl-malonylhexoside	3.11	621.1	Positive	621.1	287.4	Anthocyanin	1.03	+
Myricetin *O*-hexoside	3.5	480.1	Negative	479.1	435.3	Flavone	<1	----
Apigenin -glucuronide	2.88	446.076	Negative	445.07	269.2	Flavone	3.96	+++
Apigenin 7-*O*-glucoside (Cosmosiin)	4.11	432.1	Negative	431.097	269.044	Flavone	1.33	+
Apigenin 7-*O*-neohesperidoside (Rhoifolin)	4.4	580.1792	Negative	579.178	135	Flavone	1.91	+
Apigenin *O*-malonylhexoside	4.36	518.0	Negative	519	282.1	Flavone	1.23	+
Apigenin 7-rutinoside (Isorhoifolin)	4.42	578.1636	Positive	579.164	271.1	Flavone	<1	----
Chrysoeriol 5-*O*-hexoside	3.96	462.1	Negative	463.123	285.2	Flavone	1.13	+
Luteolin *O*-rutinoside	3.59	594.2	Positive	595.2	271.7	Flavone	<1	----
Luteolin 7-*O*-glucoside (Cynaroside)	3.61	448.101	Positive	449.1	287.1	Flavone	3.64	+++
Luteolin *O*-hexosyl-*O*-gluconic acid	3.85	625.1	Negative	624.1	285.3	Flavone	2.25	++
Luteolin *O*-malonylhexoside	4.15	534.0	Positive	535	301.7	Flavone	<1	----
Luteolin	4.91	286.2363	Negative	285	133.1	Flavone	<1	----
Quercetin *O*-hexoside	3.45	464.3	Positive	465.3	447.7	Flavonol	<1	----
Quercetin-3-*O*-rutinoside (Rutin)	3.51	610.5175	Positive	609.153	300.1	Flavonol	<1	----
Quercetin *O*-hexosyl-*O*-malonylhexoside	2.83	712.14	Positive	713.1	303.05	Flavonol	<1	----
Quercetin-3-*O*-malonylglucoside	3.93	550.1031	Negative	549.089	505.099	Flavonol	1.01	+
Quercetin-3-β-*O*-galactoside	4.26	464.1	Positive	465.1	303.6	Flavonol	<1	----
Quercetin 3-*O*-glucoside (Isotrifoliin)	3.72	464.096	Negative	463	301.1	Flavonol	1.61	+
Chrysoeriol *O*-malonylhexoside	4.43	548.1	Positive	549.1	286.7	Flavone	<1	----
Chrysin *O*-hexoside	4.67	416.2	Positive	417.2	167.5	Flavone	<1	----
Chrysoeriol *O*-ferulic acid	4.12	476.1	Positive	477.1	286.8	Flavone	<1	----
Chrysoeriol 7-*O*-rutinoside	3.92	608.2	Positive	609.2	325.8	Flavone	<1	----
Chrysoeriol *O*-hexoside	4.14	462.2	Positive	463.2	287.7	Flavone	<1	----
Chrysoeriol *O*-Glucuronic acid	4.12	476.1	Negative	475.1	299	Flavone	<1	----
Chrysin *O*-malonylhexoside	5.24	502.0	Positive	503	485.1	Flavone	<1	----
Chrysoeriol	5.6	300.0634	Negative	299.063	284	Flavone	<1	----
Isorhamnetin *O*-acetyl-hexoside	4.22	520.1	Negative	519.1	314.2	Flavone	<1	----
Tricin di-*O*-hexoside	3.49	654.1	Positive	655.1	316.2	Flavone	<1	----
Genistein (4′,5,7-Trihydroxyisoflavone)	5.45	270.2369	Negative	269.053	151	Flavone	<1	----
Tangeretin	6.32	372.3686	Positive	373.121	343	Flavone	<1	----
Eriodictyol	4.85	288.2522	Negative	287.063	151	Flavanone	<1	----
Geranyl acetate	5.91	196.28	Positive	197	161.5	Vitamins	1.92	+
Cinnamic acid	5.22	148.1586	Negative	147.052	61.8	Hydroxycinnamoyl derivatives	<1	----
Quinic acid	1.97	192.16658	Negative	191.1	85.1	Quinate and its derivatives	2.12	++
Gallic acid *O*-hexoside	1.18	332	Negative	331	313.7	Benzoic acid derivatives	1.04	
Gallic acid	1.81	170.1195	Negative	169.022	125.1	Benzoic acid	2.41	++
Benzamidine	1.31	120.154	Positive	121	77	Phenylpropanoids	<1	----
protocatechuic acid	2.42	154.027	Negative	153.1	109.1	Catechin derivatives	<1	----
Neochlorogenic acid (5-*O*-Caffeoylquinic acid)	2.53	354.1087	Positive	355.1	145.4	Quinate and its derivatives	3.16	+++
Chlorogenic acid (3-*O*-Caffeoylquinic acid)	2.69	354.0951	Negative	353.095	191.1	Quinate and its derivatives	3.05	+++
3,5-Dimethoxy-4-hydroxycinnamic acid	2.51	223.085	Negative	222.1	163	Amino acid derivatives	1.15	+
Coumaric acid *O*-glucoside	2.8	326	Negative	325	119.2	Hydroxycinnamoyl derivatives	1.23	+
Caffeic acid *O*-glucoside	3.03	342.0867	Negative	341.0876	179.033	Hydroxycinnamoyl derivatives	3.09	+++
Sinapoyl *O*-hexoside	3.27	403.155	Positive	404.155	175.4	Phenolamides	<1	----
6-Hydroxymethylherniarin	3.1	206.1	Positive	207.1	147.4	Hydroxycinnamoyl derivatives	<1	----
Caffeic acid	3.14	180.0423	Negative	179.042	134.1	Hydroxycinnamoyl derivatives	3.11	+++
5-*O*-p-Coumaroylquinic acid	3.18	338.0	Negative	337	275.8	Quinate and its derivatives	<1	----
1-*O*-β-d-Glucopyranosyl sinapate	3.1	386.1	Negative	385.1	223.2	Hydroxycinnamoyl derivatives	<1	----
Syringic acid *O*-glucoside	2.34	360.1	Negative	359.1	197.1	Benzoic acid derivatives	<1	----
1-*O*-Feruloyl quinic acid	2.94	368.1	Positive	369.1	207.5	Quinate and its derivatives	1.25	+
Coumaroylquinic acid	3.43	338.0	Negative	337	275.8	Quinate and its derivatives	2.19	++
Esculin (6,7-Dihydroxycoumarin-6-glucoside)	2.73	340.1	Negative	339.1	177.1	Coumarins	<1	----
p-Coumaric acid	3.71	164.158	Negative	163.047	119	Hydroxycinnamoyl derivatives	3.11	+++
Protocatechuic acid *O*-glucoside	4.99	316.0	Negative	315.071	153.018	Catechin derivatives	<1	----
Ferulic acid	3.92	193.1766	Negative	193.058	134.1	Hydroxycinnamoyl derivatives	2.31	++
Naringenin 7-*O*-neohesperidoside (Naringin)	3.38	580.1792	Positive	581.1	449.2	Flavanone	<1	----
Selgin *O*-malonylhexoside	3.45	564.1	Positive	565.1	317.8	Flavone	<1	----
Luteolin 6-C-glucoside	3.47	448.1	Positive	449.1	300	Flavone C-glycosides	<1	----
Luteolin C-hexoside	3.32	448.1	Positive	449.1	329.6	Flavone C-glycosides	1.04	+
Luteolin 8-C-hexosyl-*O*-hexoside	3.67	610.2	Positive	611.2	299.8	Flavone C-glycosides	<1	----
Chrysoeriol C-hexosyl	3.31	462.1	Positive	463.1	301.8	Flavone C-glycosides	<1	----
Nobiletin	3.89	402.1	Positive	403.1	373.1	Flavone	<1	----
lactucopicrin	5.26	410.1366	Negative	409.137	213.1	Others	<1	----
lactucin	3.38	276.2845	Negative	275.1	213	Others	<1	----
Eriodictyol 7-*O*-glucoside	3.75	450.3928	Negative	449	151	Flavone C-glycosides	<1	----

“+”, “++”, “+++” stands the semi quantitative content of phytochemicals.

**Table 2:** The content of mineral elements composition (mg/g DW) in six red-lettuce leaves.

	Cultivar	S-1	S-2	B-1	B-2	S-3	B-3
Ions							
**Ca**		8.275 ± 0.0760 e	8.3375 ± 0.4904 e	9.0875 ± 0.0661 d	11.2167 ± 0.0711 c	14.7915 ± 0.1909 a	14.25 ± 0.0250 b
**Cu**		0.0148 ± 0.0002 a	0.0148 ± 0.0001 a	0.0157 ± 0.0001 a	0.0149 ± 0.0001 a	0.0152 ± 0.0002 a	0.0151 ± 0.0002 a
**Fe**		0.1842 ± 0.0040 a	0.1631 ± 0.0022 b	0.1625 ± 0.0022 b	0.1850 ± 0.0057 a	0.1638 ± 0.0078 b	0.1846 ± 0.0058 a
**K**		53.1667 ± 0.2887 b	44.6250 ± 4.0292 e	55.8750 ± 0.1250 a	50.3750 ± 0.3307 c	47.4167 ± 0.4732 d	42.7083 ± 0.5052 e
**Mg**		4.6917 ± 0.0505 e	4.6667 ± 0.0289 e	5.8875 ± 0.0217 d	6.7500 ± 0.0331 c	8.6583 ± 0.1778 a	8.5292 ± 0.0315 b
**Mn**		0.0301 ± 0.0002 d	0.0379 ± 0.0008 a	0.0384 ± 0.0002 a	0.0291 ± 0.0001 e	0.0323 ± 0.0008 c	0.0348 ± 0.0001 b
**Na**		1.7083 ± 0.1512 dc	1.4983 ± 0.1118 d	2.5500 ± 0.0976 a	2.0458 ± 0.1553 b	1.8875 ± 0.0125 cb	1.8417 ± 0.1697 cb
**P**		4.0500 ± 0.0500 c	4.6000 ± 0.1083 a	4.6750 ± 0.0125 a	4.1750 ± 0.0331 b	3.3333 ± 0.0473 d	3.3208 ± 0.0072 d
**Zn**		0.0545 ± 0.0003 c	0.0584 ± 0.0004 b	0.0661 ± 0.0006 a	0.0300 ± 0.0001 e	0.0350 ± 0.0009 d	0.0360 ± 0.0010 d

Values (mean ± SD, *n* = 4) of the same ions followed by different lowercase letters (a, b, c, d, e) indicate significant difference (*p* < 0.05).

**Table 3:** Antioxidant potency composite index of flavonoids compounds in six red lettuces.

Cultivar	DPPH Index ^a^	ABTS Index ^a^	T-AOC-FRAP Index ^a^	Antioxidant Potency Composite Index ^b^
S-1	78.49	62.09	46.31	62.30
S-2	39.33	21.12	31.62	30.69
B-1	48.58	29.01	23.40	33.66
B-2	100.00	100.00	97.25	99.08
S-3	71.87	61.58	49.97	61.14
B-3	98.97	96.44	100.00	98.47

DPPH: free radical scavenging properties by 1,1-diphenyl-2-picrylhydrazyl radical; ABTS: 2,2′-azinobis-(3-ethylbenzothiazoline-6-sulfonic acid) radical. Antioxidant index score = [(sample score/best score) × 100], T-AOC-FRAP: stands using the ferric reducing activity of plasma (FRAP) assay to measure total antioxidant activity (T-AOC). ^a^ averaged of the DPPH, ABTS, T-AOC-FRAP assay for the antioxidant potency composite index, ^b^ averaged of the DPPH and ABTS tests for the antioxidant potency composite index.

## Supplementary Materials

Supplementary materials can be found at http://www.mdpi.com/1422-0067/19/4/1165/s1.

## References

[1] Llorach R., Martínez-Sánchez A., Tomás-Barberán F.A., Gil M.I., Ferreres F. (2008). Characterisation of polyphenols and antioxidant properties of five lettuce varieties and escarole. Food Chem..

[2] Nicolle C., Cardinault N., Gueux E., Jaffrelo L., Rock E., Mazur A., Amouroux P., Rémésy C. (2004). Health effect of vegetable-based diet: Lettuce consumption improves cholesterol metabolism and antioxidant status in the rat. Clin. Nutr..

[3] Urszula Z., Micha Ś., Anna J. (2014). Effect of abiotic elicitation on main health-promoting compounds, antioxidant activity and commercial quality of butter lettuce (*Lactuca sativa* L.). Food Chem..

[4] Kim M.J., Moon Y., Tou J.C., Mou B., Waterland N.L. (2016). Nutritional value, bioactive compounds and health benefits of lettuce (*Lactuca sativa* L.). J. Food Compos. Anal..

[5] Mulabagal V., Ngouajio M., Nair A., Zhang Y.J., Gottumukkala A.L., Nair M.G. (2010). In vitro evaluation of red and green lettuce (*Lactuca sativa*) for functional food properties. Food Chem..

[6] Siti Azima A.M., Noriham A., Manshoor N. (2014). Anthocyanin content in relation to the antioxidant activity and colour properties of Garcinia mangostana peel, *Syzigium cumini* and *Clitoria ternatea* extracts. Int. Food Res. J..

[7] Han K.H., Sekikawa M., Shimada K., Hashimoto M., Hashimoto N., Noda T., Tanaka H., Fukushima M. (2006). Anthocyanin-rich purple potato flake extract has antioxidant capacity and improves antioxidant potential in rats. Br. J. Nutr..

[8] Li H., Deng Z., Zhu H., Hu C., Liu R., Young J.C., Tsao R. (2012). Highly pigmented vegetables: Anthocyanin compositions and their role in antioxidant activities. Food Res. Int..

[9] Ferreres F., Gil M.I., Castaner M., Tomasbarberan F.A. (1997). Phenolic metabolites in red pigmented lettuce (*Lactuca sativa*). Changes with minimal processing and cold storage. J. Agric. Food Chem..

[10] Garcia C.J., García-Villalba R., Garrido Y., Gil M.I., Tomás-Barberán F.A. (2016). Untargeted metabolomics approach using UPLC-ESI-QTOF-MS to explore the metabolome of fresh-cut iceberg lettuce. Metabolomics.

[11] Becker C. (2016). Flavonoids and phenolic acids in lettuce: How can we maximize their concentration? And why should we?. Acta Hortic..

[12] Khoo H.E., Azlan A., Tang S.T., Lim S.M. (2017). Anthocyanidins and anthocyanins: Colored pigments as food, pharmaceutical ingredients, and the potential health benefits. Food Nutr. Res..

[13] Fang J. (2015). Classification of fruits based on anthocyanin types and relevance to their health effects. Nutrition.

[14] Wang L., Li H., Yang S., Ma W., Liu M., Guo S., Zhan J., Zhang H., Tsang S.Y., Zhang Z. (2016). Cyanidin-3-o-glucoside directly binds to ERα36 and inhibits EGFR-positive triple-negative breast cancer. Oncotarget.

[15] Viacava G.E., Roura S.I., Berrueta L.A., Iriondo C., Gallo B., Alonso-Salces R.M. (2017). Characterization of phenolic compounds in green and red oak-leaf lettuce cultivars by UHPLC-DAD-ESI-QToF/MS using MSE scan mode. J. Mass Spectrom..

[16] Park S., Arasu M.V., Lee M.K., Chun J.H., Seo J.M., Lee S.W., Al-Dhabi N.A., Kim S.J. (2014). Quantification of glucosinolates, anthocyanins, free amino acids, and vitamin C in inbred lines of cabbage (*Brassica oleracea* L.). Food Chem..

[17] Marín A., Rubio J.S., Martínez V., Gil M.I (2009). Antioxidant compounds in green and red peppers as affected by irrigation frequency, salinity and nutrient solution composition. J. Sci. Food Agric..

[18] Kim D.E., Shang X., Assefa A.D., Keum Y.S., Saini R.K. (2018). Metabolite profiling of green, green/red, and red lettuce cultivars: Variation in health beneficial compounds and antioxidant potential. Food Res. Int..

[19] Baslam M., Morales F., Garmendia I., Goicoechea N. (2013). Nutritional quality of outer and inner leaves of green and red pigmented lettuces (*Lactuca sativa* L.) consumed as salads. Sci. Hortic..

[20] Tsao R., Khanizadeh S., Dale A. (2006). Designer fruits and vegetables with enriched phytochemicals for human health. Can. J. Plant Sci..

[21] Morales-Soto A., García-Salas P., Rodríguez-Pérez C., Jiménez-Sánchez C., de la Luz Cádiz-Gurrea M., Segura-Carretero A., Fernández-Gutiérrez A. (2014). Antioxidant capacity of 44 cultivars of fruits and vegetables grown in Andalusia (Spain). Food Res. Int..

[22] Heo H.J., Kim Y.J., Chung D., Kim D.O. (2007). Antioxidant capacities of individual and combined phenolics in a model system. Food Chem..

[23] Sofo A., Lundegårdh B., Mårtensson A., Manfra M., Pepe G., Sommella E., Nisco M.D., Tenore G.C., Campiglia P., Scopa A. (2016). Different agronomic and fertilization systems affect polyphenolic profile, antioxidant capacity and mineral composition of lettuce. Sci. Hortic..

[24] Alarcón-Flores M.I., Romero-González R., Martínez Vidal J.L., Garrido Frenich A. (2016). Multiclass Determination of Phenolic Compounds in Different Varieties of Tomato and Lettuce by Ultra High Performance Liquid Chromatography Coupled to Tandem Mass Spectrometry. Int. J. Food Prop..

[25] Romani A., Pinelli P., Galardi C., Sani G., Cimato A., Heimler D. (2002). Polyphenols in greenhouse and open-air-grown lettuce. Food Chem..

[26] Llorach R., And F.A.T., Ferreres F. (2004). Lettuce and Chicory Byproducts as a Source of Antioxidant Phenolic Extracts. J. Agric. Food Chem..

[27] Plumb R., Mazzeo J.R., Grumbach E.S., Rainville P., Jones M., Wheat T., Neue U.D., Smith B., Johnson K.A. (2007). The application of small porous particles, high temperatures, and high pressures to generate very high resolution LC and LC/MS separations. J. Sep. Sci..

[28] Jaiswal R., Kiprotich J., Kuhnert N. (2011). Determination of the hydroxycinnamate profile of 12 members of the Asteraceae family. Phytochemistry.

[29] Arzu A., Vural G. (2009). Effect of various anti-browning agents on phenolic compounds profile of fresh lettuce (L. *sativa*). Food Chem..

[30] Becker C., Klaering H.P., Kroh L.W., Krumbein A. (2014). Cool-cultivated red leaf lettuce accumulates cyanidin-3-*O*-(6”-*O*-malonyl)-glucoside and caffeoylmalic acid. Food Chem..

[31] Fallovo C., Rouphael Y., Cardarelli M., Rea E., Battistelli A., Colla G. (2009). Yield and quality of leafy lettuce in response to nutrient solution composition and growing season. Int. J. Food Agric. Environ..

[32] Mengjiao X., Yingyan H., Xiaoxiao Q., Zhifan S., Shuangxi F. (2017). Study of nutritional quality and antioxidant activity in varieties of leaf lettuce. J. Beijing Univ. Agric..

[33] Song J., Zhao M., Liu X., Zhu Y., Hu X., Chen F. (2013). Protection of cyanidin-3-glucoside against oxidative stress induced by acrylamide in human MDA-MB-231 cells. Food Chem. Toxicol..

[34] Kong J.M., Chia L.S., Goh N.K., Chia T.F., Brouillard R. (2003). Analysis and biological activities of anthocyanins. Phytochemistry.

[35] Bub A., Watzl B., Heeb D., Rechkemmer G., Briviba K. (2001). Malvidin-3-glucoside bioavailability in humans after ingestion of red wine, dealcoholized red wine and red grape juice. Eur. J. Nutr..

[36] Manach C., Morand C., Gilizquierdo A., Bouteloupdemange C., Rémésy C. (2003). Bioavailability in humans of the flavanones hesperidin and narirutin after the ingestion of two doses of orange juice. Eur. J. Clin. Nutr..

[37] Erlund I., Meririnne E.G., Aro A. (2001). Plasma kinetics and urinary excretion of the flavanones naringenin and hesperetin in humans after ingestion of orange juice and grapefruit juice. J. Nutr..

[38] Serafini M., Bugianesi R., Salucci M., Azzini E., Raguzzini A., Maiani G. (2002). Effect of acute ingestion of fresh and stored lettuce (*Lactuca sativa*) on plasma total antioxidant capacity and antioxidant levels in human subjects. Br. J. Nutr..

[39] Suffness M., Pezzuto J.M., Hostettmann K. (1990). Assays related to cancer drug discovery. Methods in Plant Biochemistry: Assays for Bioactivity.

[40] John K.M., Jung E.S., Lee S., Kim J.-S., Lee C.H. (2013). Primary and secondary metabolites variation of soybean contaminated with Aspergillus sojae. Food Res. Int..

[41] Lee S., Do S.-G., Kim S.Y., Kim J., Jin Y., Lee C.H. (2012). Mass spectrometry-based metabolite profiling and antioxidant activity of Aloe vera (*Aloe barbadensis* Miller) in different growth stages. J. Agric. Food Chem..

[42] Qin X., Xing Y.F., Zhou Z., Yao Y. (2015). Dihydrochalcone Compounds Isolated from Crabapple Leaves Showed Anticancer Effects on Human Cancer Cell Lines. Molecules.

[43] Dai Q., Yin Q., Zhao Y., Guo R., Li Z., Ma S., Lu N. (2015). III-10, a newly synthesized flavonoid, induces cell apoptosis with the involvement of reactive oxygen species-mitochondria pathway in human hepatocellular carcinoma cells. Eur. J. Pharmacol..

[44] Strober W. (2001). Trypan blue exclusion test of cell viability. Curr. Protoc. Immunol..

[45] Abel S.D.A., Baird S.K. (2017). Honey is cytotoxic towards prostate cancer cells but interacts with the MTT reagent: considerations for the choice of cell viability assay. Food Chem..

