# Supplementary material for: Beneficial Phytochemicals with Anti-Tumor Potential Revealed through Metabolic Profiling of New Red Pigmented Lettuces (Lactuca sativa L.)
Source: Int J Mol Sci. 2018 Apr 11;19(4):1165. doi: 10.3390/ijms19041165 (PMC5979491; doi:10.3390/ijms19041165)
Supplement: Supplementary file 1 [file ijms-19-01165-s001.pdf]

Table S1. Significant difference metabolites content in six red pigmented lettuces cultivars. Values (mean ± SD, n=3) of the same compound followed by different lowercase letters indicate significant difference ($p<0.01$).

**Compounds**	**Area ×10^5**					
	**S-1**	**S-2**	**S-3**	**B-1**	**B-2**	**B-3**
Malvidin 3-O-glucoside (Oenin)	1.41±0.04b	1.22±0.06b	0.92±0.04c	1.04±0.04c	2.59±0.13a	2.15±0.09a
Cyanidin 3-O-glucosyl-malonylglucoside	16.89±0.51b	4.56±0.23c	15.85±0.63b	4.35±0.17c	26.40±1.32a	18.70±0.75b
Cyanidin-3,5-O-diglucoside (Cyanin)	1.46±0.04b	0.93±0.04c	1.36±0.05b	0.41±0.02d	3.14±0.16a	1.52±0.06b
Apigenin -glucuronide	4.12±0.1b	2.55±0.13c	1.68±0.07d	3.25±0.13b	7.21±0.36a	5.03±0.20b
Luteolin 7-O-glucoside (Cynaroside)	1.12±0.06d	1.55±0.12d	0.68±0.002e	2.23±0.20c	6.25±0.41a	4.61±0.29b
Luteolin O-hexosyl-O-gluconic acid	0.06±0.001b	0.05±0.001b	0.08±0.001b	0.04±0.001b	0.15±0.002a	0.03±0.001b
Quinic acid	49.55±2.4b	32.00±1.62c	25.05±1.98c	26.10±1.01c	75.20±3.71a	53.40±2.46b
Gallic acid	121.50±3.64b	87.25±4.36c	87.60±3.50c	86.15±3.45c	158.80±7.91a	132.50±5.37b
Neochlorogenic acid (5-O-Caffeoylquinic acid)	49.55±1.47b	32.00±1.60c	25.05±1.00c	26.10±1.04c	75.20±3.76a	53.40±2.14b
Chlorogenic acid (3-O-Caffeoylquinic acid)	577.50±17.23b	607.5±30.36a	549.50±21.98c	565.00±22.45b	620.00±31.00a	543.00±21.71c
Caffeic acid O-glucoside	29.65±0.89b	19.60±0.98c	19.30±0.77c	20.80±0.83c	44.35±2.22a	43.55±1.74a
Caffeic acid	181.00±5.43b	185.5±9.38b	150.15±6.01d	141.45±5.65e	248.50±12.43a	160.25±6.41c
Coumaroylquinic acid	0.65±0.02b	0.57±0.03c	0.52±0.02c	0.63±0.03b	0.72±0.04a	0.61±0.02b
p-Coumaric acid	3.88±0.12b	4.03±0.20b	3.64±0.15c	2.91±0.12d	5.17±0.26a	3.62±0.14c
Ferulic acid	0.88±0.026b	0.62±0.03c	1.08±0.04a	0.66±0.03c	1.31±0.07a	0.75±0.03b

Peak Area (Area) represents the relative content of the metabolites.
